# Learning in ensembles of proteinoid microspheres

**DOI:** 10.1098/rsos.230936

**Published:** 2023-10-11

**Authors:** Panagiotis Mougkogiannis, Andrew Adamatzky

**Affiliations:** Unconventional Computing Laboratory, UWE, Bristol, UK

**Keywords:** thermal proteins, proteinoids, microspheres, unconventional computing

## Abstract

Proteinoids are thermal proteins which form microspheres in water in the presence of salt. Ensembles of proteinoid microspheres exhibit passive nonlinear electrical properties and active neuron-like spiking of electrical potential. We propose that various neuromorphic computing architectures can be prototyped from the proteinoid microspheres. A key feature of a neuromorphic system is a learning. Through the use of optical and resistance measurements, we study mechanisms of learning in ensembles of proteinoid microspheres. We analyse 16 types of proteinoids study and their intrinsic morphology and electrical properties. We demonstrate that proteinoids can learn, memorize and habituate, making them a promising candidate for novel computing.

## 1. Introduction

Proteinoids, or thermal proteins, are produced by heating amino acids to their melting point and initiation of polymerization to produce polymeric chains, which are swelled in aqueous solution forming hollow structures known as microspheres [1,2]. Being able to form spontaneously from simple chemical mixes, proteinoids are speculated to have played a crucial role in the early phases of Earth’s creation of life [1–3]. A remarkable feature of proteinoids, which earned them name of proto-neurons [2,4,5], is that the microspheres maintain a membrane potential of 20 to 70 mV without any stimulating current [6] and exhibit oscillations of the electrical potential strikingly similar to action potential of neurons [7,8]. These oscillations can be observed for several days and even weeks [4,6]. In [9], we proposed to use ensembles of the proteinoid microspheres as neuromorphic unconventional computing devices. A key feature of a neuromorphic system is a learning. A learning in physical systems is a formation of conductive pathways [10]. In order to trigger the creation of conducting pathways, the proteinoid network must be subjected to electrical stimuli, e.g. by applying a potential difference between two electrodes inserted in the ensemble of proteinoid microspheres. The voltage should lead to reorganization of the proteinoid molecules and generating preferable routes for the flow of electrical current. Rearrangements of proteinoids molecules might create structures morphologically similar to neural dendrites and axons paths [11–15].

**Figure 1.**
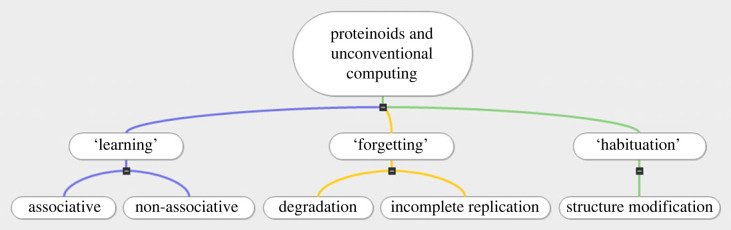
The scheme illustrates how proteinoids can be used in unconventional computing, with a specific emphasis on their capacity for learning and memory, as well as their habituation to stimuli and the ability to adapt their behaviour accordingly. Proteinoid degradation, imperfect replication, structural alteration and incomplete replication are all proven to play a role in the ‘learning’, ‘forgetting’ and ‘habituation’ processes.

We look into the conductivity and habituation properties of proteinoids to determine their potential in electronics. Figure 1 emphasizes the three important parameters of learning, forgetting and habituation, which show the potential of proteinoids in unconventional computing [16,17]. In particular, figure 1 demonstrates how proteinoids can be employed to keep track of data and adjust to new conditions.

Superior efficiency, reduced power consumption and increased connectivity with other devices are just some of the benefits that can be realized by using novel semiconductor materials instead of more traditional options. Third-generation semiconductor materials based on SiC and GaN, and two-dimensional semiconductor materials based on graphene, are two examples of innovative semiconductor materials [18]. Proteinoids are materials with the ability to ‘learn’ from their surroundings, ‘forget’ and ‘habituate', and alter their resistance accordingly. We will investigate how innovative semiconductors can benefit from these features to improve their performance and reliability.

Associative learning is a form of learning that takes place when a novel semiconductor learns to link an input with a stimulus [19,20]. By recognizing and responding to patterns of input and output, proteinoids can fine-tune their resistance. An innovative semiconductor, for instance, might be programmed to increase its resistance in response to stressors like high temperatures and humidity.

When proteinoids are subjected to the same stimuli over and over again, a sort of learning known as ‘non-associative’ learning takes place [21–23]. A proteinoid might, for instance, be programmed to lower its resistance in response to particular sounds or frequencies. Proteinoids can employ this form of learning to adjust to new environments. Proteinoids can experience several forms of forgetting [24] depending on how they lose their training data: degradation or incomplete replication. This forgetting can be exploited to reduce the robustness and lifespan of new semiconductors in many settings. An example would be instructing a proteinoid to forget what it has learnt after a particular period of time has passed. Useful for preventing new semiconductors from being weighed down by antiquated data, this form of forgetting can be implemented.

To break down a proteinoid into smaller molecules, a certain amount of voltage, known as the ‘critical voltage', must be applied [25]. A proteinoid’s composition and structure establish its critical resistance and critical voltage. Proteinoids rich in sulfur-containing amino acids tend to have higher critical resistance and critical voltage than those rich in polar amino acids. Greater molecular-weight proteinoids also have higher critical resistance and voltage values than smaller molecular-weight proteinoids.

The development of proteinoid-powered devices, such as biosensors and unconventional computing, increases the significance of this discovery. To elucidate the temporal behaviour of proteinoids, the critical resistance and voltage of the proteinoid network will be examined. This study also offers information on the connection between the structure of the proteinoid network and its electrical conductivity. The findings of this study can be used for the creation of proteinoid networks with improved functionality.

## 2. Methods

L-glutamic acid (L-Glu), L-aspartic acid (L-Asp), L-histidine (L-His), L-lysine acetate (L-Lys), L-phenylalanine (L-Phe) and L-arginine (L-Arg) with a purity of greater than 98% (Sigma-Aldrich) and poly(L-lactic acid) from (Polysciences) have been used. The gold-coated proteinoids were examined using an FEI Quanta 650 field emission scanning electron microscopy, and all chemicals employed were used as is. Imaging samples that have been gold-coated prior to imaging will produce higher-quality results by minimizing the negative impact of charging effects on the images. Proteinoids have been prepared and characterized in a similar fashion to what has been described previously in [26].

The absorbance of proteinoids at room temperature was determined using a UV-visible spectrometer (Jenway 7200). The recommended calibration range of 200–800 nm was employed, and the device was calibrated accordingly. Absorbance data for the proteinoids were measured at 0.5 nm intervals.

A Keithley 2450 SourceMeter was used to measure the electrical properties of the proteinoids. All readings were taken at room temperature of 19−20°C. The electrical resistance of proteinoid samples was measured using a four-probe technique with collinearly aligned platinum–iridium probes spaced 10 mm apart. The probes were immersed in the proteinoid solutions and a constant DC current of 1 mA was applied using a Keithley 2450 SourceMeter. The voltage drop between the inner probes was measured with a resolution of 0.1 μV to determine the resistance according to Ohm’s Law (R = V/I). The Keithley 2450 SourceMeter instrumentation used provides high precision multi-channel I–V measurement capabilities (Style Standard Append Mode 1, Fill Mode 1, Capacity 900000, Count 26084, Base Time Seconds 1678819153, Base Time Fractional 0.2468152). Finally, the I–V response was examined and linear ohmic behaviour was observed, as expected for low-contact resistance.

Matlab was used to analyse and plot the data from the UV visible spectrometer and the source meter. We looked for relationships between the absorbance of proteinoids and their current, voltage and resistance by comparing these quantities.

## 3. Results

### 3.1. Structural characteristics and optical band gap

**Figure 2.**
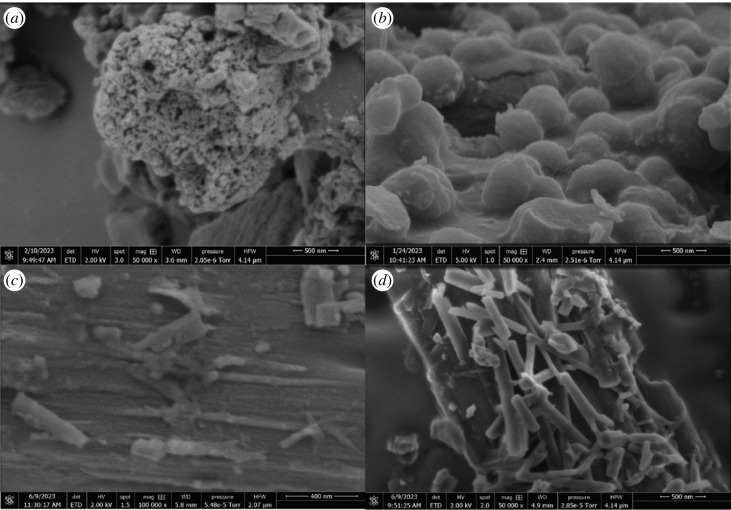
This scanning electron microscope image depicts the proteinoids (*a*) L-Phe:L-Arg and (*b*) L-Glu:L-Phe:L-Asp as spherical structures prior to the application of an electrical stimulus. Scanning electron micrographs (*c*) and (*d*) of aggregated proteoids following repetitive electrical pulse stimulation (magnification of 500 000×). The presence of protruding dendrite- and axon-like formations from protenoid structures is indicative of habituation. Scale bar equals 400 nm in (*c*), 500 nm in (*a*), (*b*) and (*d*).

Before electrical stimulation, the protenoid aggregates did not have distinct neural-like morphologies. Instead, they appeared mostly as uniform spheres (figure 2*a*,*b*). Upon repeated electrical pulsing, extended dendrite- and axon-like projections emerged from the protenoid structures (figure 2*c*,*d*). The presence of these structures that resemble neurons offers concrete evidence of habituation effects in protenoid systems. This finding aligns with their demonstrated ability to learn. The remodelling of protenoid aggregates, which results in the growth of dendrite- and axon-like structures when stimulated, indicates that they have the ability to adapt structurally in a biomimetic way, resembling natural neurons.

**Figure 3.**
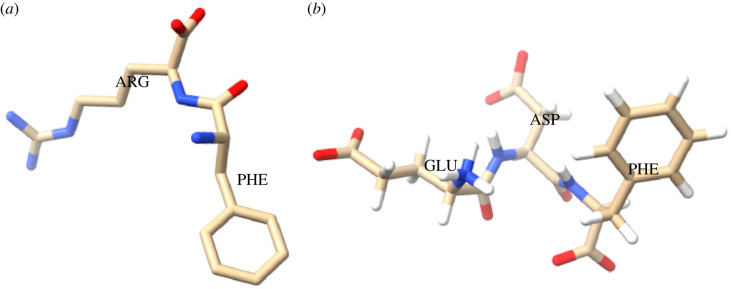
(*a*) L-Phe:L-Arg and (*b*) L-Glu:L-Phe:L-Asp proteinoids, with nitrogen shown in blue, oxygen in red and carbon in yellow, respectively. The L-Phe:L-Arg structure is made up of just those two amino acids, while the L-Glu:L-Phe:L-Asp structure adds glutamic acid to the scene. Structures generated in Chimera.

The polypeptide structure is a stable helical form held together by the strand due to the hydrogen bonds between amino acids. Due to the strong interactions between amino acid side chains, proteinoids take the shape of a sphere when they adopt this helical conformation. Two proteinoids, L-Phe:L-Arg and L-Glu:L-Phe:L-Asp, have their molecular structures depicted in figure 3.

The precise arrangement of amino acids in the proteinoid is also a contributing factor in the production of perfectly round microspheres. Combinations of aromatic (phenylalanine) and charged (arginine and aspartic acid) amino acids can be found in proteinoids like L-Phe:L-Arg and L-Glu:L-Phe:L-Asp. In contrast to the hydrophilic regions formed by arginine and aspartic acid, which are attracted to water molecules, the hydrophobic regions formed by the aromatic amino acid phenylalanine surround the proteinoids. By balancing out the hydrophilic and hydrophobic regions, the proteinoids take a spherical shape due to the overall hydrophobic surface tension.

**Figure 4.**
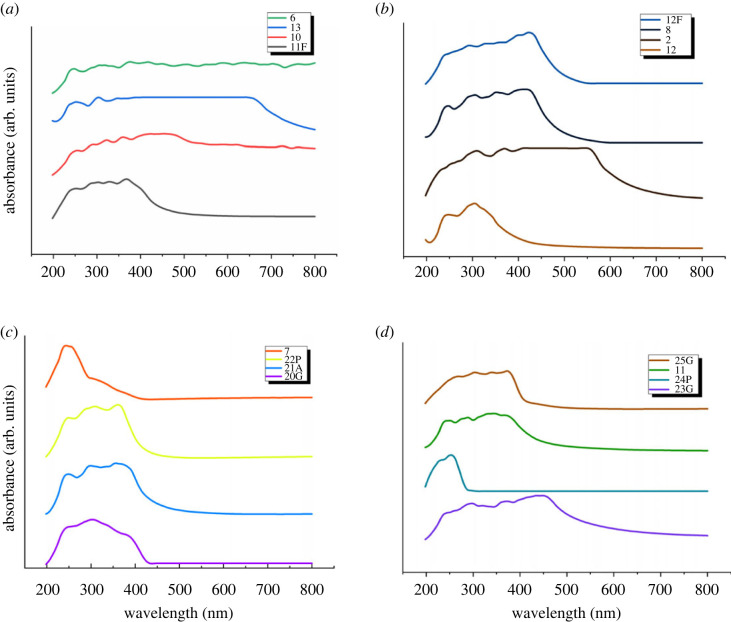
The picture depicts the ultraviolet (UV) spectra of 16 distinct proteinoid molecules spanning from 198 to 800 nm in wavelength. Diverse absorbance readings across the spectrum suggest that several distinct proteinoid molecules are present in the sample.

Sixteen distinct proteinoids—determined by various combinations of amino acids—were analysed via their UV spectra (figure 4). The spectrum was measured between 198 and 800 nm. Each proteinoid was discovered to have its own spectral fingerprint (table 1). The presence of aromatic amino acids in the proteinoids was shown by the presence of a peak in the spectra near 280 nm.

**Table 1.** The maximum absorption wavelength *λ*_max_ and the optical band gap *E*_*g*_ for each proteinoid. The experiment shows that the maximum absorption wavelength is the reverse of the optical band gap. This means that the proteinoids can absorb light at different wavelengths.

proteinoid	*λ*_max_ (nm)	*E*_*g*_ (eV)	code of exp.
L-Glu:L-Asp	553	2.33	2
L-Glu:L-Phe:L-His	423	2.93	8
L-Lys:L-Phe:L-His	303	4.10	13
L-Glu:L-Phe	421	2.95	10
L-Glu:L-Asp:L-Lys	343	3.62	11
L-Glu	302	4.11	12
L-Glu:L-Phe:PLLA	367	3.38	11F
L-Lys:L-Phe-L-His:PLLA	427	2.90	12F
L-Lys:L-Phe:L-Glu	243	5.10	7
L-Glu:L-Phe	375	3.31	25G
L-Asp	358	3.46	21A
L-Phe:L-Lys	366	3.39	22P
L-Glu:L-Arg	300	4.13	20G
L-Phe	254	4.88	24P
L-Glu:L-Asp:L-Pro	458	2.71	23G
L-Glu:L-Asp:L-Phe	374	3.32	6

What is known as a material’s optical band gap is the energy difference between its highest valence band and its lowest conduction band. As such, it is a key factor in determining how a material will behave electrically and optically. It can be used to determine whether a material is an insulator or a semiconductor, and it can also be used to foretell whether or not a certain material will absorb or emit light. The optical bandgap was determined from Tauc plots based on the following equation:

3.1 $$\alpha h\nu =\text{const}{\left(h\nu -E_{g}\right)}^{n},$$

where *α* is the absorption coefficient, *hν* is the photon energy of incident light, *E*_g_ is the optical bandgap and *n* characterizes the optical absorption process. Tauc plots were then created, showing the relationship between (*αhν*)^2^ and photon energy *hν*, where *α* represents the absorption coefficient. The optical bandgap was determined by extrapolating the linear region of the plot until it intersected with the *x*-axis. The method has been widely used to estimate bandgaps based on optical absorbance spectra [27,28].

Several absorption peaks can be seen in proteinoids, as seen in figure 4 This is because of the molecular structure, which makes it possible for distinct parts to absorb light of different colours.

### 3.2. Voltage-induced conductive pathways

We apply a DC voltage between 0 and 3 V and measure the resulting change in resistance, denoted by *R*_1_. Once this is done, the sample’s resistance, represented by *R*_2_, can be calculated. Typically, a lower result for *R*_2_ than *R*_1_ indicates that the sample’s resistance has decreased. It is necessary to repeat this method for a variety of proteinoids to get an accurate reading of their resistance levels.

**Figure 5.**
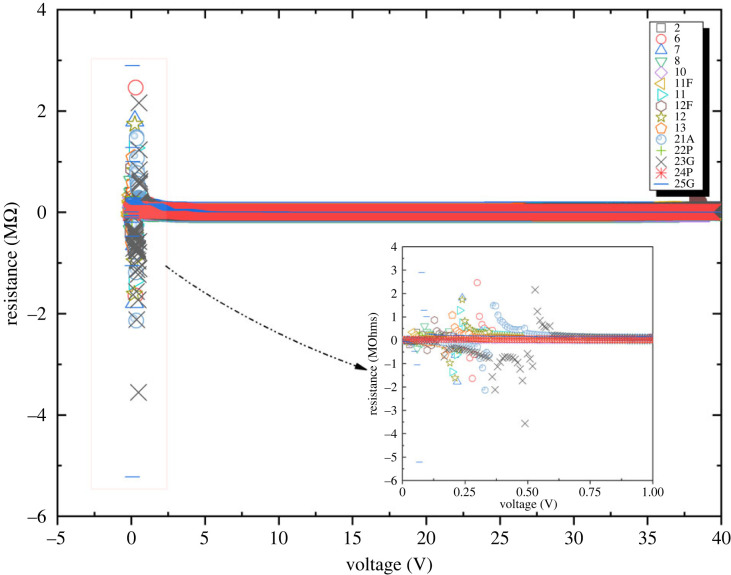
Resistance versus voltage measurements of 16 types of proteinoids. The data show a wide range of resistance values across different proteinoids in response to increasing voltage.

Taking into account voltage 0.08 V and resistance 2.9 MΩ, the proteinoid L-Glu:L-Phe (code 10) appears to have the highest resistance of any proteinoid. This conclusion is supported by the data presented in figure 5. The graph demonstrates that L-Glu:L-Phe is more resistant to a given voltage than the other proteinoids. If compared with the resistance of other proteinoids, which is between 0.0067 and 2.464 MΩ, this is a huge increase.

Surprisingly high resistance is seen in proteinoids at near-zero voltages, but this resistance drops exponentially with increasing voltage up to around 40 V, where it then plateaus.

As can be observed in figure 5, at low-voltage proteinoids occasionally show a negative resistance, meaning that an increase in voltage results in a drop in current.

The resistance of proteinoids increases when voltage between 0 and 0.25 or 0.5 V applied but starts to increase when the applied voltage exceeds 5 V, as seen in electronic supplementary material. The expansion rate of L-Asp is the highest (code 21A). The ‘proteinoids threshold’ describes this phenomenon: electrostatic interactions between charged peptide units in proteinoids polypeptides are responsible for the proteinoids threshold. As the peptides establish stronger inter-molecular connections at low voltages, the resistance of the proteinoids rises. The energy needed to force the current through the proteinoid rises in proportion to the square of the increase in resistance. Yet, as the voltage increases, the electrostatic connections lessen, resulting in a decrease in the proteinoid’s resistance. As a result of the reduced resistance, less energy is needed to drive the current through the proteinoid [29].

### 3.3. ‘Forgetting’: resistance rises in the absence of the stimuli

Here, we investigate the potential application of ‘forgetting’ in unconventional computing by observing how turning off stimulation alters the resistance along the proteinoid conductive channel. The outcomes of certain computations, e.g. machine learning, genetic algorithms, evolutionary computing, can be affected by the ability to ‘forget’.

Our methodology for analysing the findings involves taking readings of the resistance of the conductive pathways at regular intervals and comparing them with the readings taken before the stimulation began. This information is used to assess memory loss—represented as a conductivity decline—across a range of proteinoids.

To visualize how a device’s current (*I*) and voltage (*V*) are related, an *I* − *V* curve can be drawn. The parameters that can be derived from an *I* − *V* curve vary with the component or device being studied. For instance, the slope of an *I* − *V* graph can be used to determine a resistor’s conductance. Finding the point where the diode’s *I* − *V* curve begins to grow sharply allows one to calculate the diode’s threshold voltage.

The theory of varistors is fundamental to the investigation of proteinoids conductivity and other electrical phenomena. It asserts that the amount of current flowing through a material is equal to the ratio of the applied voltage (*V*) and a constant, *K*_0_. The law (equation (3.2))

3.2 $$I=\frac{K_{0}}{V^{\alpha }},$$

expresses this connection between these two variables. In order to comprehend how substances react when subjected to an electric field, the varistor theory is a crucial idea. The extent to which the electrical characteristics of a material change in response to a change in voltage is described by this relationship (equation (3.2)). The material’s intrinsic constant *K*_0_ characterizes the level of its nonlinearity.

Many different types of materials, including semiconductors, ferroelectrics and insulators, are predicted by the varistor theory. Semiconductors are widely employed in electronics because of their malleability in electrical applications. The resistance of these components changes in response to an external voltage. Materials with a permanent electric dipole moment are known as ferroelectric materials and have several practical uses. Insulators prevent electrical current from flowing because their composition prevents electrons from freely moving [30]. Using equation (3.3), we can calculate the stimulation length in seconds (see electronic supplementary material).

3.3 $$\text{stimulus length}=\frac{\text{voltage range}}{\text{each stimulus duration}},$$

with the voltage range being 3 V. The time of dropping resistance measures how long it takes for the electrical simulation to reach its peak resistance before it starts to drop.

The term ‘quantile’ is used to describe the equal division of a dataset. The 99.5 percentile is the value below which 99.5% of the data points lie; the 75.0 percentile is the value below which 75.0% of the data points lie; and the 0.0 percentile is the lowest value in the dataset.

**Figure 6.**
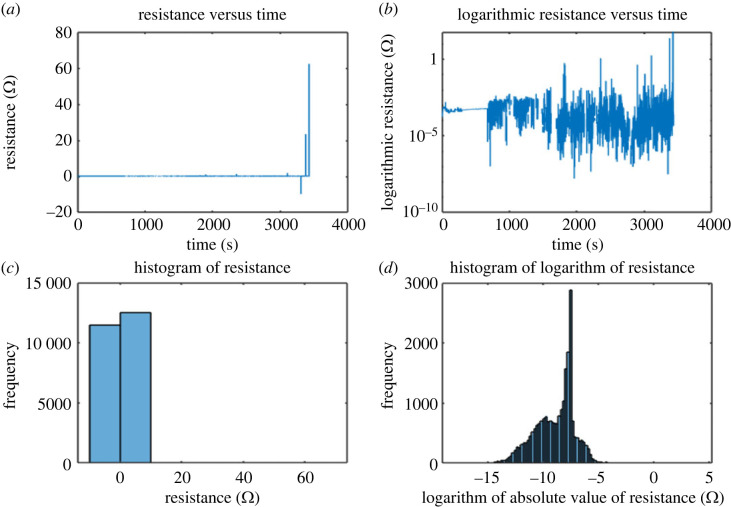
This figure shows (*a*,*b*) the resistance with code 2 over time and (*c*,*d*) the distribution of resistance measurements. The upper 95% mean of resistance is 9038.5 Ω and the lower 95% mean is 1930.7 Ω.

Proteinoid 2 (see codes of the proteinoid in table 1) resistance quantile measurements (figure 6) reveal some intriguing trends as follows. In total, 100% of the total resistance was reached at a value of 62.1 MΩ. Although most resistance values were far less than the maximum value, the 99.5% quantile value of 4360 Ω demonstrates this. When compared with the highest possible value, the median of 7.197 Ω is quite low. For a perfect percentage, the lowest value was −9 984 000 Ω. These findings demonstrate that the resistance values of proteinoid 2 varied widely, with some extreme cases present. There appeared to be a fair deal of fluctuation in the strength of proteinoid 2, as the majority of resistance readings were significantly lower than the maximum.

Using a quantile regression analysis, the range of resistance values was calculated for proteinoid 6, with quantiles of 100% 416544.1 Ω, 99.50% 6934.5 Ω, 97.50% 3984.6 Ω, median 0.15 Ω and 0.01% quantile −7503 421 Ω. According to the findings, the levels of resistance can be anywhere from −750.3 Ω to +41654.1 Ω. The data also reveal that 416544.1 Ω is a significantly greater resistance value than the rest of the values in the range. This indicates that there is a very high resistance number that stands in stark contrast to the rest of the data.

**Figure 7.**
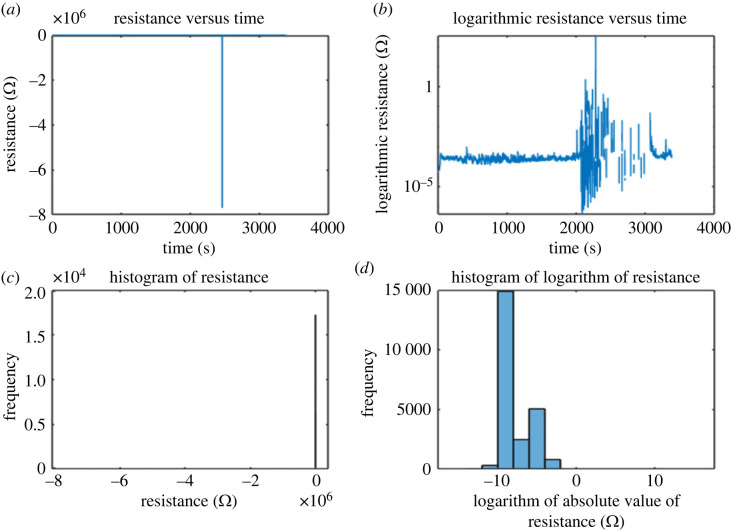
(*a,b*) Resistance versus time from proteinoid 7 under constant potential 20 V and (*c,d*) histogram of resistance values with quantiles at 100%, 99.5%, 97.5%, 90%, median and 0.0%. The resistance values in Ωs are 362 430 464, 10111, 593.2, 339, 261.4, 217.1 and −7.7 × 10^12^, respectively. The graph of proteinoid 7 resistance versus time shows time passing in an arrow-like fashion.

Figure 7 shows that the 100% percentile resistance value is 362 MΩ. Resistance of 10 111 Ω represents the 99.5% quantile. The resistance value at the 97.5 percentile is 593.2 Ω , at the 90 percentile it is 339 Ω, and at the 75 percentile it is 261.4 Ω . There is a wide range of resistance values, with a median of 217.1 Ω and a lowest value of $-7.7\times 10^{9}\;M\Omega$ (at the 0.0 percentile). This suggests a wide variation in resistance ratings for proteinoid 7 (see codes in table 1).

Compared with the other quantiles, the greatest resistance value of 362 MΩ is extremely high, suggesting that only a small fraction of the sample has such a high resistance value. The inclusion of a few samples with exceptionally low resistance values could possibly account for the fact that the lowest resistance value is much lower than the other quantiles, at $-47.7\times 10^{9}\;M\Omega$.

For proteinoids 11F, 12F, 13, 22P, 23G, 25G and 11 (see codes in table 1), an examination of the resistance as a function of time reveals several intriguing tendencies (see figures in electronics supplementary material). The most striking pattern here is the sudden rise in resistance between 0 and 100 s. Following this boost, the resistance returns to a more manageable level and stays there until the 5000 s mark, at which point it begins to gradually grow again. Between 0 and 100 s, resistance increases dramatically, most likely because proteinoid microspheres are interacting with one another and forming more complex structures. This interaction makes the microspheres more robust to external influences, resulting in an increase in resistance. This boost in resistance is followed by a period of stability that lasts for around 5.5 min. It is possible that the formation of increasingly complex structures has contributed to this steady rise in resistance.

Table 2 demonstrates a large dispersion in resistance data histogram quantiles across 16 proteinoids. Proteinoid resistance was found to be maximal at the 100% level, and to diminish with each succeeding quantile. From the data of resistance of table 2, we can see that the resistance range of protenoids 2, 6, 7, 8, 10, 11, 11F, 12F, 12, 13, 20G, 21A, 22P, 23G, 24P and 25G is between −9.98 MΩ and 62.1 MΩ, −7.5 MΩ and 0.42 MΩ, $-7.71\times 10^{+12}\;M\Omega$ and 362.4 MΩ, 84.7 Ω and 0.76 MΩ, 112.7 Ω and 0.0044 MΩ, −0.43 MΩ and 0.303 MΩ, −7.33 MΩ and 7.2 MΩ, −66.9 MΩ and 25.3 MΩ, 36.9 Ω and 1801 Ω, $-2.14\times 10^{+8}\;M\Omega$ and $2.4\times 10^{+8}\;M\Omega$, −0.34 MΩ and 0.148 MΩ, 90.3 Ω and 3239 Ω, −2.4 MΩ and 1.06 MΩ, $-2.07\times 10^{+8}\;M\Omega$ and $9.8\times 10^{+7}\;M\Omega$, 74.8 Ω and 3638.8 Ω, respectively. Increased resistance can be attributed to the proteinoid’s more intricate structure, which features both polar and non-polar parts. Another cause for wide range of resistance values is due to the increased quantity of cross-linking between the microspheres, which increases the strength and stiffness of the proteinoid structure.

**Table 2.** Quantiles of resistance distribution (Ω) for 16 different proteinoids.

	*R*		*R*	*R*	*R*	*R*
	100%	*R*	75.0%	50%	25.0%	0.0%
code	maximum	99.5%	quartile	median	quartile	minimum
of exp.	(Ω)	(Ω)	(Ω)	(Ω)	(Ω)	(Ω)
2	62 084 437	4359.9654	474.32786	7.1971977	−2785.324	−9984000
6	416544.06	6934.5387	1.2881781	0.1536745	−26.63374	−7503421
7	362430464	10110.566	261.35351	217.14726	−242.3689	−7.71 × 10^12^
8	76091.049	6183.5833	2325.6716	1418.6132	692.87616	84.70032
10	4404.9648	2688.7351	1628.779	1129.6275	554.34378	112.67969
11	303036.25	8691.1443	1391.5009	946.35925	628.69757	−431974.8
11F	7.411 × 10^12^	24619418	586.01653	195.45464	−4082.635	−1.56 × 10^13^
12F	7186565.8	107251.93	2041.8851	741.79247	−2451.287	−7331591
12	25315922	286739.73	9459.7533	5205.7754	2926.6472	−66917508
13	1801.1524	1630.706	831.3615	430.28272	202.04338	36.918475
20G	2.389 × 10^11^	149585.92	39.122431	−0.157974	−52.63574	−2.14 × 10^11^
21A	148750.47	8159.6149	−392.6154	−1801.791	−3559.802	−341609
22P	3239.7874	2331.8801	831.36735	391.21415	261.61789	90.301161
23G	1061668.3	20931.568	3288.7011	1871.6031	376.94064	−2409099
24P	9.823 × 10^10^	18445.175	3.7424821	0.5117157	−3.252959	−2.07 × 10^11^
25G	3638.8308	2393.2651	1433.5912	740.98793	379.97653	74.836721

**Figure 8.**
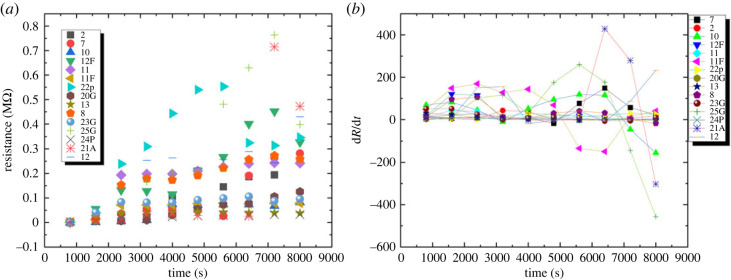
(*a*) The variation in resistance over time for 16 different proteinoids. Codes of the proteinoids are given in table 1. (*b*) Resistance derivation versus time. This graph depicts the resistance rise over the time for 16 distinct proteinoids.

The results demonstrated that the 16 proteinoids’ resistance increased with response to time (figure 8). Resistance of proteinoids varied from 0.00104 MΩ to 0.764 MΩ at the start, and peaked at 0.057 MΩ after 8000 s.

A first-order differential equation is one alternative mathematical model for describing the resistance’s time-dependent evolution. One application of this type of equation is modelling the time-dependent resistance. Forms that the equation can take are

3.4 $$\frac{dR}{dt}=k\times R+b.$$

Current versus voltage characteristics (see electronic supplementary material) were measured 10 times at constant voltages between 0 and 40 V, and it was found that the slope of the I–V curve decreases with each additional measurement, indicating higher resistance as the number of measurements grows.

It is the material’s physical qualities that are responsible for the drop in resistance. A rise in voltage energizes the material’s electrons, facilitating their freer movement and lowering the material’s resistance. The I–V curve, which displays the resistance of a device, flattens out when more I–V readings are taken in a row, indicating an increase in resistance.

One possible explanation for the increased resistance is that the material itself was altered by the heating process. In response to an increase in voltage, a material’s internal temperature rises, potentially resulting in a reduction in the material’s resistance due to increased atomic vibration. To further illustrate the results, figure 8 clearly demonstrates the resistance versus time increase for 16 different proteinoids. Resistance at time *t* is denoted by *R*, the rate of change of resistance over time is denoted by *k*, and the offset or bias term is denoted by *b*. It is possible to estimate *k* and *b* using experimental data in order to fit the model to the observed behaviour.

**Figure 9.**
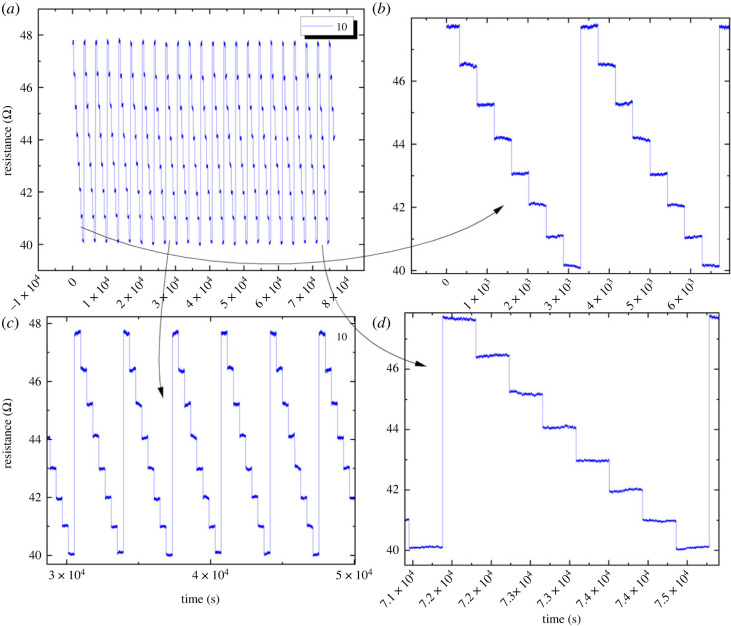
This plot displays the resistance habituation with time for proteinoid 10, in response to a voltage arbitrary function with a frequency of 280 μHz and an amplitude of 2 mV_*pp*_.

Resistance adapted from 40.1 to 47.8 Ω when an arbitrary wave function with a frequency of 280 µHz and an amplitude of $2\;{\mathrm{mV}}_{\;pp}$ was applied, as depicted in figure 9. Using a voltage function, the graph shows that the resistance of the material steadily increased from 40.1 to 47.8 Ω, suggesting that it had become accustomed to the applied voltage.

### 3.4. Habituation of proteinoids through ‘learning’ and ‘forgetting’ cycles

The habituation of proteinoids is studied by subjecting them to stimuli at timed intervals and then comparing pre- and post-stimulation resistance readings. This is accomplished by first measuring the proteinoids’ resistance (*R*) without subjecting them to any stimulation. At times *T*_1_, *T*_2_ and so on, up to *T*_*n*_, stimuli are introduced. The resistance of the proteinoids, designated *R*_*i*_, is measured again after each stimulation. Habituation is demonstrated if and only if it can be shown that $R_{T_{1}}$ is smaller than $R_{T_{2}}$, and $R_{T_{2}}$ is less than $R_{T_{3}}$, and so on.

**Figure 10.**
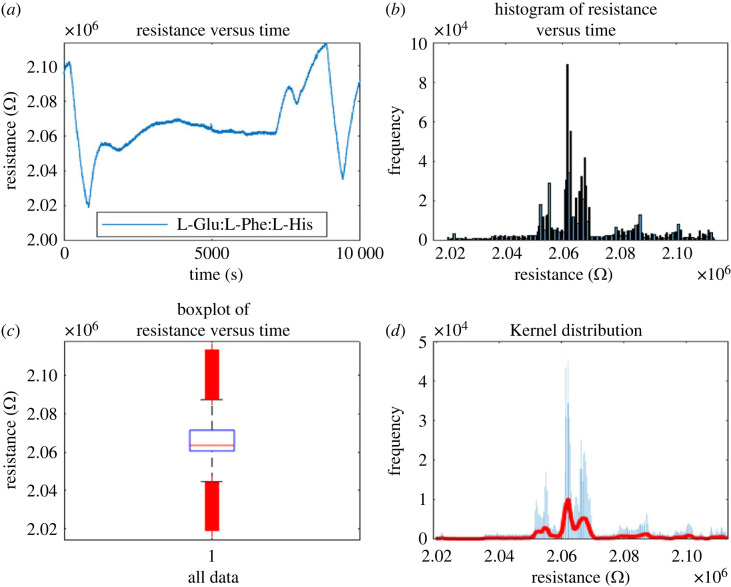
(*a*) Measurements of proteinoid code 8 resistance versus time using open circuit potentiometry. (*b*) Distribution of resistance levels for proteinoid 8 over time as shown by a histogram. (*c*) Average resistance is found to be 2067342.3 Ω, with a median of 2063572 Ω and a standard variation of 17364.2 Ω, as shown by the boxplot. Inter-quartile range is 10812 Ω. (*d*) Data-driven fitting of a kernel distribution function.

**Figure 11.**
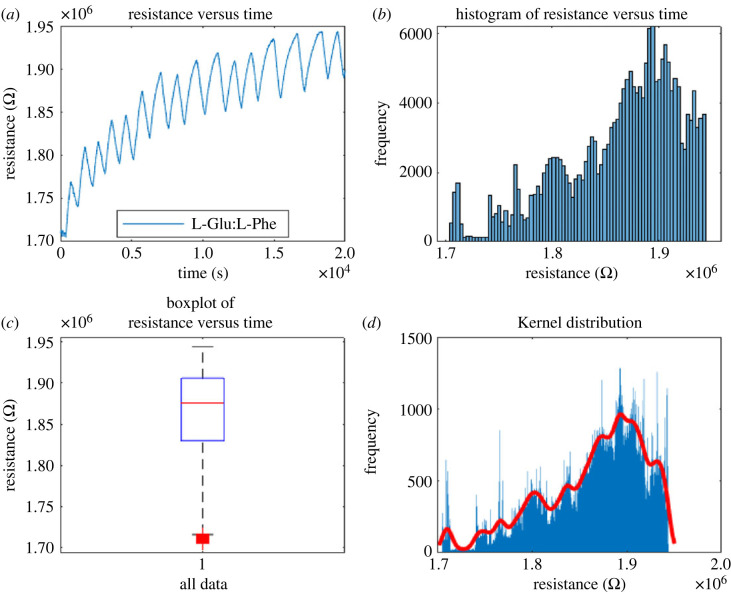
(*a*) The relationship between resistance and time was measured using open circuit potentiometry for proteinoid code 25G. (*b*) Distribution of resistance data for proteinoid 8 over time, a histogram showing the relationship between resistance and time. (*c*) The boxplot identifies mean value of resistance 1863778.3 Ω, median 1875762.2 Ω, standard deviation 55364.1 Ω and inter-quantile range 75956.5 Ω. (*d*) Data fit using the kernel distribution function.

**Figure 12.**
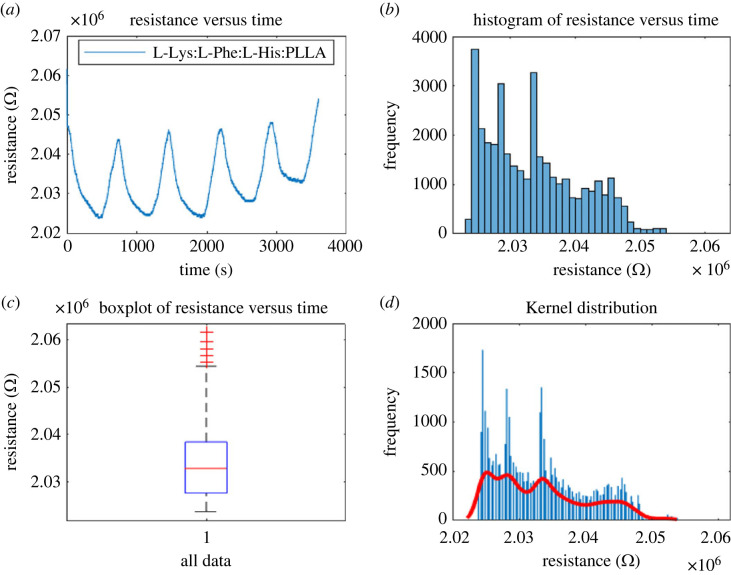
(*a*) The relationship between resistance and time was measured using open circuit potentiometry for proteinoid code 12F. (*b*) Distribution of resistance data for proteinoid 8 over time, a histogram showing the relationship between resistance and time. (*c*) The boxplot identifies mean value of resistance 2033420.4982 Ω, median 2032772 Ω, standard deviation 7082.6487 Ω and inter-quantile range 10812 Ω. (*d*) Data fit using the kernel distribution function.

Proteinoids with codes 8, 25 G and 12F (see codes in table 1) have been analysed for their habituation characteristics in figures 10, 11 and 12. According to the findings, proteinoid 25G has superior habituation characteristics compared with proteinoid 8. The average resistance of 25G proteinoids is 1863778.3 Ω, the median is 1875762.2 Ω, the standard deviation is 55364.1 Ω and the inter-quantile range is 75956.5 Ω. Furthermore, there is a reasonable match between the results and the kernel distribution for proteinoid 25G.

The resistance versus time data was fitted using the kernel distribution, which is a non-parametric depiction of the probability density function. The likelihood of witnessing a specific resistance value at a specific time was faithfully represented by the kernel distribution. This helped us make sense of our data and learn more about the correlation between resistance and time.

The findings show that proteinoid 25G is better suited for uses like unconventional computing because it is less likely to cause habituation than proteinoid 8. The findings also imply that protenoid 25G could be used in other contexts, such as cell-based treatments, where resistance to habituation is desirable.

## 4. Discussion

In laboratory experiments, we demonstrated that proteinoids can ‘remember’, ‘forget’ and ‘habituate’, thus presenting essential features of living nervous systems.

Real-world memory is the recall of previously learned information. But, the ability to do this is not unique to humans; it can be recreated using biological molecules. Proteinoids can show signs of memory through a process called associative learning, which allows them to store and retrieve information. The proteinoids’ ability to self-assemble and reproduce underlies their memory and retrieval capabilities. By experiencing a stimulus and responding to it in the future, a proteinoid can acquire the ability to learn. This property of proteinoids makes them suitable for use as memory in unconventional computing systems.

Forgetting is the process of erasing one’s memories of the past through time. We have shown that proteinoids gradually lose the information—in the form of resistance capacity [31]—when stimulation stops. This aids proteinoids’ ability to focus on the most important data in unconventional computing by filtering out the noise. This makes it less likely that the proteinoids-based neuromorphic computing devices [9] will become overloaded with data.

**Figure 13.**
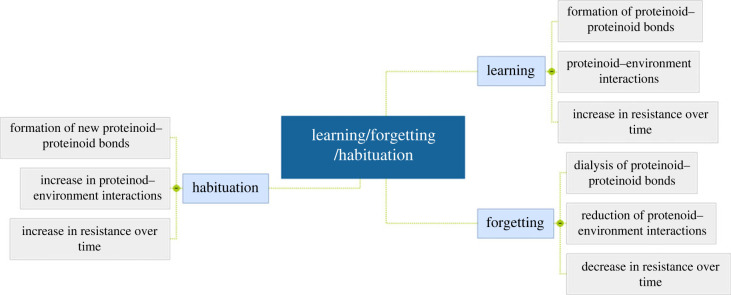
This mind map demonstrates the mechanism of proteinoids’ learning, forgetting and habituation. Proteinoids learn by making new connections to external stimuli, forget by weakening and severing old connections, and get accustomed by learning to disregard extraneous stimuli. This process is exemplified by the fluctuation in resistance over time.

Figure 13 outlines the mechanism of learning, forgetting and habituation, with a focus on the variation of proteinoid resistance across time.

Other non-trivial responses of proteinoids observed in experiments are discussed below.

In the experiments, we found that for low voltages resistance becomes negative. This happens because of how proteinoids are built; under particular conditions, such as low voltage, they can form complexes that cause this effect. Many proteinoid molecules connect with one another in these complexes, and as the voltage is raised, the proteinoids bind more securely to one another, thereby decreasing the current. Because of this drop in current, negative resistance is present. The use of bioelectronic devices is one area where negative resistance might be advantageous. With this quality, designers can craft oscillators and filters with very distinct electrical profiles, allowing them to execute very specialized functions. The decreased current draw of the negative resistance results in a lower power requirement for operation of the device, making this proteinoid feature useful for designing energy-efficient circuits. By harnessing negative resistance, it is possible to amplify weak signals from biological sensors or stimulators that monitor or regulate various bodily functions such as inflammation, neural signalling and cardiac output. It also helps in generating oscillations or pulses that can modulate biological processes such as gene expression, hormone secretion and circadian rhythms. Furthermore, the utilization of negative resistance enables the creation of compact, high-speed, low-voltage digital logic circuits for bioelectronics. This not only reduces power requirements but also minimizes heat generation. Negative resistance offers various opportunities to enhance the performance, capabilities and miniaturization of bioelectronic technologies that are specifically designed to interface with biological systems through electrical signals [32–35].

We found that L-Glu:L-Phe is more resistant to a changing voltage than the other proteinoids. This indicates that L-Glu:L-Phe is a better option for voltage regulation since it is more resistant to voltage at these levels. Several theories suggest that the structure, composition and presence of particular functional groups in the L-Glu:L-Phe proteinoid are responsible for this enhanced resistance. For instance, in comparison with the other studied proteinoids, L-Glu:L-Phe has a larger proportion of hydrophobic moieties among its amino acid residues. The L-Glu:L-Phe proteinoid also has a larger concentration of carboxyl and amide groups, which may account for its enhanced resistance. Although it is folded into an *α*-helical shape, this proteinoid is smaller than the others we tested.

A high resistance was recorded in proteinoids at near-zero voltages, but this resistance drops exponentially with increasing voltage up to around 40 V. This discovery is crucial because it proves proteinoids may be used to build robust electrical circuits. As proteinoids can function at low voltages while still providing the required resistance, they make a great choice for designing dependable electric circuits. Additionally, proteinoids exhibit a consistent resistance even when the applied voltage exceeds 40 V. This opens the door for the usage of proteinoid circuits in many other contexts, including ones with varying voltage requirements.

As a result of their enhanced resistance, proteinoids can be used for a variety of purposes, including as electrical insulation, electrical shielding and thermal insulation. The signal from biological systems can be detected and amplified using proteinoids because they are employed as a substrate in biosensors. The higher resistance of proteinoids is significant for these applications, since it helps to ensure that the signal is not degraded by electrical interference. In other words, the proteinoids’ increased resistance to voltage is a direct result of their having gradually formed a more stable structure over time.

Organic, biocompatible, self-assembling, programmable and responsive to external stimuli, proteinoids are ideal for use as unconventional computer materials. They are well suited for imitating neural networks and artificial brains due to their peculiar characteristics, such as action-potential-like spiking of electrical potential.

How can proteinoids use their electrical potential spikes to remember and retrieve information?

Primitive proteins, or proteinoids, may store information and retrieve it via electrical potential spikes. Information storage and processing in these organisms have been extensively explored because they provide such an intriguing paradigm for early life forms seen in prebiotic chemistry. Proteinoids have the power to construct memories through a variety of mechanisms, including memory formation and consolidation, adaption to changing environmental conditions, habituation and sensitivity to stimuli and forgetting rate and memory decay.

When it comes to proteinoids, how exactly do memories get stored in their microspheres brain?

Proteinoids use a wide variety of chemical processes and electrical potentials to build and consolidate memories. Using electrical potentials, proteinoids are able to store information within their structures, which in turn sets off a cascade of processes that results in the creation of memory. It is believed that electrostatic forces acting on the proteinoids are the mediating factor in the creation and consolidation of memories.

When faced with new stimuli and environments, how do proteinoids respond?

Proteinoids also possess the remarkable ability to adapt to novel stimuli and situations in their environment. Proteinoids can change their memory and learn from new ‘experiences’ to adapt to their surroundings. Because of this, they are able to adapt to different stimuli and environments with relative ease and efficiency.

How fast do proteinoids ‘forget’ things, and what causes their memories to fade over time?

A proteinoid’s forgetting rate and memory loss can be affected by a number of variables, including the stimulus’s type, length and electrical potential. While proteinoids can retain and retrieve information for a long time, the memory is more likely to fade the longer the stimulus last. The rate at which memories fade and are forgotten can also be affected by the voltage of the electrical potentials.

How can proteinoids show habituation and sensitization to familiar and unfamiliar stimuli?

Habituation and sensitization to novel or unfamiliar stimuli are also behaviours that proteinoids can display. It is possible to become more sensitive to a stimulus or to respond more strongly to it with repeated exposure, a process called sensitization, as opposed to being accustomed to or desensitized to it, as is the case with habituation. Proteinoids rely on this sort of behaviour so that they can swiftly and accurately adapt to their surroundings.

Thus, employing proteinoids as memory devices in organic electronic unconventional computing devices has both positive and negative aspects. Proteinoids’ ability to rapidly store and process massive amounts of data is a major benefit.

## 5. Conclusion

So far, the studies of proteinoids’ cognitive abilities have shown encouraging results. Indeed, proteinoids have been shown to be capable of learning, memory and forgetting. They have the ability to build conductive channels and microspheres, as well as the capacity to form memories and adaptation to environmental stimuli. This is a major development for the future of biomimetic and unconventional computing. More study and experimentation are needed, but it is possible that one day organic computer systems will mimic proteinoids’ learning abilities. Ultimately, proteinoids have been shown to be an important and useful tool in the study of novel semiconductors, and they may be the key to enabling innovative approaches to unconventional computing.

## Data Availability

This web page contains the data for the publication: https://doi.org/10.5061/dryad.mgqnk994r [36]. The data are provided as a CSV file, which contains the information associated with the results of the study. The data are provided in electronic supplementary material [37].

## References

[1] Harada K, Fox SW. 1958 The thermal condensation of glutamic acid and glycine to linear peptides. J. Am. Chem. Soc. **80**, 2694-2697. (10.1021/ja01544a027)

[2] Fox SW. 1992 Thermal proteins in the first life and in the ‘mind-body’ problem. In *Evolution of information processing systems* (ed. K Haefner, pp. 203–228. Berlin, Germany: Springer. (10.1007/978-3-642-77211-5_12)

[3] Fiore M. 2019 The origin and early evolution of life: prebiotic chemistry. Life **9**, 73. (10.3390/life9030073) 31547394 PMC6789705

[4] Yu B, Pappelis A, Sikes CS, Fox SW. 1994 Evidence that the protocell was also a protoneuron. *Orig. Life Evol. Biosph.* **24**.

[5] Fox SW et al. 1995 Experimental retracement of the origins of a protocell. J. Biol. Phys. **20**, 17-36. (10.1007/BF00700418)

[6] Przybylski AT. 1985 Excitable cell made of thermal proteinoids. BioSystems **17**, 281-288. (10.1016/0303-2647(85)90044-9) 4052590

[7] Ishima Y, Przybylski AT, Fox SW. 1981 Electrical membrane phenomena in spherules from proteinoid and lecithin. BioSystems **13**, 243-251. (10.1016/0303-2647(81)90004-6) 7248487

[8] Przybylski AT, Stratten WP, Syren RM, Fox SW. 1982 Membrane, action, and oscillatory potentials in simulated protocells. Naturwissenschaften **69**, 561-563. (10.1007/BF00396351) 7162535

[9] Adamatzky A. 2021 Towards proteinoid computers: hypothesis paper. Biosystems **208**, 104480. (10.1016/j.biosystems.2021.104480) 34265376

[10] Xu Q, Jin L, Li C, Kuddannayai S, Zhang Y. 2018 The effect of electrical stimulation on cortical cells in 3D nanofibrous scaffolds. RSC Adv. **8**, 11 027-11 035. (10.1039/C8RA01323C) PMC9079102 35541524

[11] Lico DT. 2023. The biological and structural organization of the squid brain. In *Animal models and experimental research in medicine*. IntechOpen.

[12] Glock C, Biever A, Tushev G, Nassim-Assir B, Kao A, Bartnik I, tom Dieck S, Schuman EM. 2021 The translatome of neuronal cell bodies, dendrites, and axons. Proc. Natl Acad. Sci. USA **118**, e2113929118. (10.1073/pnas.2113929118) 34670838 PMC8639352

[13] Ludwig PE, Reddy V, Varacallo M. 2022 Neuroanatomy, neurons. In *StatPearls* [Internet]. Treasure Island, FL: StatPearls Publishing.

[14] Paus T. 2022 Tracking development of connectivity in the human brain: axons and dendrites. Biol. Psychiatry **93**, 455-463. (10.1016/j.biopsych.2022.08.019) 36344316

[15] Bromberg Y, Aptekmann AA, Mahlich Y, Cook L, Senn S, Miller M, Nanda V, Ferreiro DU, Falkowski PG. 2022 Quantifying structural relationships of metal-binding sites suggests origins of biological electron transfer. Sci. Adv. **8**, eabj3984. (10.1126/sciadv.abj3984) 35030025 PMC8759750

[16] Tayur S. 2021 Unconventional computing: applications, hardware, algorithms. *Quantum Computing*. (10.1287/orms.2021.01.14)

[17] Ziegler M. 2020 Novel hardware and concepts for unconventional computing. *Sci. Rep.* **10**, 11843. (10.1038/s41598-020-68834-1) PMC7366690 32678249

[18] Stahl FA. 2003 The emergence of semiconductors: nineteenth century modern physics. Am. J. Phys. **71**, 1170-1173. (10.1119/1.1596175)

[19] Li Z, Fattah A, Timashev P, Zaikin A. 2022 An account of models of molecular circuits for associative learning with reinforcement effect and forced dissociation. Sensors **22**, 5907. (10.3390/s22155907) 35957464 PMC9371404

[20] Tan J, Cheng Z, Feldmann J, N Youngblood XL, Ali UE, Wright CD, Pernice WHP, Bhaskaran H. 2020 Monadic pavlovian associative learning in a backpropagation-free photonic network. *arXiv*. (http://arxiv.org/abs/2011.14709)

[21] Mondal S et al. 2022 All-electric nonassociative learning in nickel oxide. Adv. Intell. Syst. **4**, 2200069. (10.1002/aisy.202200069)

[22] Ioannou A, Anastassiou-Hadjicharalambous X. 2018 *Non-associative Learning*, pp. 1–13. Cham, Switzerland: Springer International Publishing.

[23] Non-Associative Learning - an overview | ScienceDirect Topics. See https://www.sciencedirect.com/topics/psychology/non-associative-learning.

[24] Berry JA, Cervantes-Sandoval I, Nicholas EP, Davis RL. 2012 Dopamine is required for learning and forgetting in *Drosophila*. Neuron **74**, 530-542. (10.1016/j.neuron.2012.04.007) 22578504 PMC4083655

[25] Critical Voltage - an overview | ScienceDirect Topics. See https://www.sciencedirect.com/topics/engineering/critical-voltage.

[26] Mougkogiannis P, Adamatzky A. 2023 Low frequency electrical waves in ensembles of proteinoid microspheres. Sci. Rep. **13**, 1992. (10.1038/s41598-023-29067-0) 36737467 PMC9898556

[27] Namgung SD, Lee J, Choe IR, Sung T, Kim Y-O, Lee Y-S, Nam KT, Kwon J-Y. 2017 Increased electrical conductivity of peptides through annealing process. APL Mater. **5**, 086109. (10.1063/1.4997562)

[28] Kheirabadi NR, Chioleriob A, Phillipsa N, Adamatzky A. 2022 Learning in colloids: synapse-like ZnO + DMSO colloid. *arXiv.* (http://arxiv.org/abs/2211.00419)

[29] Wuthrich R., Abou Ziki, J.D., 2014. Micromachining using electrochemical discharge phenomenon: fundamentals and application of spark assisted chemical engraving. Norwich, NY: William Andrew.

[30] Levine JD. 1975 Theory of varistor electronic properties. Crit. Rev. Solid State Mater. Sci. **5**, 597-608. (10.1080/10408437508243517)

[31] Mougkogiannis P, Phillips N, Adamatzky A. 2023 Transfer functions of proteinoid microspheres. *arXiv*. (http://arxiv.org/abs/2302.05255)

[32] Shaali R, Doroodmand MM, Moazeni M. 2021 Diode and active negative resistance behaviors of helminth eggs as a novel identification/differentiation probe. ACS Omega **6**, 33 728-33 734. (10.1021/acsomega.1c04954) PMC8674989 34926921

[33] Khatami Y, Kang J, Banerjee K. 2013 Graphene nanoribbon based negative resistance device for ultra-low voltage digital logic applications. Appl. Phys. Lett. **102**, 043114. (10.1063/1.4788684)

[34] Song M-K et al. 2023 Tyrosine-mediated analog resistive switching for artificial neural networks. Nano Res. **16**, 858-864. (10.1007/s12274-022-4760-1)

[35] Adewuyi A, Lau WJ. 2023 Development of nanocomposite membranes for removal of pharmaceutically active compounds in water: a review. Appl. Chem. Eng. **6**, 2066. (10.24294/ace.v6i2.2066)

[36] Mougkogiannis P, Adamatzky A. 2023 Data from: Learning in ensembles of proteinoid microspheres. Dryad Digital Repository. (10.5061/dryad.mgqnk994r) PMC10565364 37830018

[37] Mougkogiannis P, Adamatzky A. 2023 Learning in ensembles of proteinoid microspheres. Figshare. (10.6084/m9.figshare.c.6884226) PMC10565364 37830018

